# “Sit Yourself Down”: Women’s Experiences of Negotiating Physical Activity During Pregnancy

**DOI:** 10.1177/1049732320909103

**Published:** 2020-03-06

**Authors:** Janelle M. Wagnild, Tessa M. Pollard

**Affiliations:** 1 Durham University, Durham, United Kingdom

**Keywords:** physical activity, pregnancy, social practice, qualitative, interviews, Europe, England

## Abstract

Approaches to understanding why physical activity (PA) tends to decline during pregnancy are generally based on individualized behavioral models, examining “barriers” or “enablers.” In contrast, we used a social practice approach to explore the ways in which women negotiate PA during pregnancy within the contexts and routines of their everyday lives. Semistructured interviews were conducted with 18 pregnant women who had been classed as being at risk of gestational diabetes. We found that leisure-time physical activities were valued as pleasurable and therapeutic, but women’s roles as employees and carers for others often constrained their opportunities for leisure-time PA. Women encountered others’ expectations that they should sit down and slow down. This surveillance was often resisted, as women relied on “listening to the body” as a way to negotiate PA. These findings have important implications for public health strategies or interventions designed to promote PA during pregnancy.

## Introduction

Physical activity (PA) during pregnancy is associated with a number of positive health outcomes, including reduced risk of gestational diabetes (GDM; DiPietro et al., 2019). Government guidelines in countries including the United Kingdom recommend that pregnant women without contraindications accumulate the same volume of PA that is recommended for nonpregnant women (and men): at least 150 minutes of moderate-intensity PA per week (Department of Health and Social Care, 2019). As PA is strongly linked with improved insulin levels and glycemic control (see Colberg et al., 2016), medical guidelines suggest that PA is particularly important for pregnant women who are either at risk for, or have been diagnosed with, GDM (Colberg et al., 2016; National Institute for Health and Care Excellence, 2015). However, PA levels have been shown to decline from prepregnancy to pregnancy (Fell et al., 2009; Pereira et al., 2007).

Understanding the low participation of pregnant women in PA has been of great interest to public health and PA researchers over the past several decades. To date, the vast majority of this work has approached this phenomenon using theoretical frameworks focused on individualized models of behavior (Thompson et al., 2017). From such theoretical standpoints, emphasis has been on factors that “influence” women’s PA during pregnancy in terms of attitudes, beliefs, motivations, intentions, barriers, and enablers (Harrison et al., 2018). For example, these studies have consistently identified physical discomfort, tiredness, lack of social support, or concerns about the safety of PA for the fetus as “barriers” to PA during pregnancy (Connelly et al., 2015; Denison et al., 2015; Evenson et al., 2009; Flannery et al., 2018; Weir et al., 2010), while weight management and easier labor have been identified as “motivators” and “benefits” (Bauer et al., 2018; Cioffi et al., 2010; Tucker & Fouts, 2016; Weir et al., 2010).

Approaching PA as a “health behavior” and using individualized models to understand it can result in an oversimplified and decontextualized understanding of how PA is (or is not) experienced and integrated into everyday life (Blue et al., 2016; Cohn, 2014). This approach surmises that behaviors are largely the outcomes of mere decision making and does not allow for an exploration of the complexities and fluidity of everyday lives and social worlds. Considering different forms of PA during pregnancy as social practices, instead of PA as a “health behavior,” can add deeper insight into how PA during pregnancy is experienced and negotiated within the contexts of women’s material and social worlds. We draw on this approach, which reorients the research focus toward understanding how PAs are situated and experienced within the complexity of everyday life, rather than focusing on decontextualized “reasons” women do or do not take part in PA during their pregnancies. This creates space to consider the meanings that may be attached to certain practices (Blue et al., 2016; Shove et al., 2012) which may be a critical component that has heretofore been absent in explorations of PA during pregnancy.

Feminist analyses of the medicalization of pregnancy and the surveillance of pregnant bodies have shown that in contemporary “Western” contexts, pregnant women are positioned as responsible for their fetus’ health (Lupton, 1999, 2012; Warin et al., 2012). They are expected to modify their practices during pregnancy to protect the “vulnerable” fetus from all potential risks and threats (Lupton, 1999, 2012) and are subjected to continuous public surveillance and critical gazes which challenge “deviant” practices (Burton-Jeangros, 2011; Greene et al., 2017; Lupton, 1999, 2012; Parsons et al., 2014; Triandafilidis et al., 2016). The way in which PA practices during pregnancy are interpreted within these discourses of risk and maternal responsibility may vary. For example, recent studies in Canada showed that pregnant women understood their own health as intimately connected to their fetus’ health, and consequently considered engaging in PA practices as important for improving fetal health through improving maternal health (Harper & Rail, 2012; Jette & Rail, 2014). On the contrary, however, concerns about the possible risks that PA might pose to the fetus date back centuries (see Green, 2002); thus PA may be avoided, or scrutinized by others, for that reason (van Mulken et al., 2016). These understandings of the value or risk associated with PA during pregnancy are likely to play an important role in women’s experiences of PA during pregnancy.

The aim of this article is to explore women’s accounts of how they experience and negotiate PA during pregnancy. We focused on the experiences of women who had been told they were at risk of developing GDM, for whom PA is considered particularly important.

## Methods

Semistructured interviews were conducted with 18 pregnant women in the third trimester of pregnancy who were recruited from two National Health Service (NHS) antenatal clinics in North East England in 2017 for a larger study and agreed to participate in this part of the study. Women were recruited into the main study when they attended the hospital for their 12-week ultrasound scan (see Wagnild et al., 2019), and participants who indicated interest in being interviewed on the study enrollment form were contacted when they reached 30 weeks’ gestation. As the larger study concerned the link between PA and GDM among women classed as high risk for GDM (Wagnild et al., 2019), all participants had at least one risk factor for GDM at the time of recruitment: high body mass index (BMI, ≥30 kg/m^2^), family history of diabetes, ethnicity associated with high diabetes prevalence, previous macrosomia, or previous GDM (National Institute for Health and Care Excellence, 2015). They had been told that they were considered at high risk of GDM at around 8 weeks’ gestation, and they were tested for GDM by oral glucose tolerance tests between weeks 24 to 28. By the time the interviews took place, all participants had been screened for GDM; four women were diagnosed with GDM and had received their diagnoses at least 1 month prior to the interview. All participants were at least 18 years of age, fluent in English, and pregnant with only one baby.

Interviews usually took place in participants’ homes or cafes, but several (*n* = 5) took place in private rooms in the hospital per the participants’ requests. In all but one case, the interviewer had already met the participant over the course of other study activities, so some rapport had already been established prior to the interview. The interview guide was agreed between the authors and was designed to ask about participants’ experiences of pregnancy, how these experiences compared to their expectations of what this pregnancy would be like, and whether there were things about their pregnancy that they didn’t expect (Supplemental File). While participants knew that the larger study focused on the link between PA and GDM, questions specifically about PA were not included in the interview schedule to allow the topic to emerge naturally (if at all) within participants’ narratives of their pregnancies; where PA was mentioned, follow-up questions probed for contextual details. The interviewer explicitly stated to the participants prior to each interview that she was not a health care professional or an expert, and that there were no “right” or “wrong” answers to any of the questions. Interviews usually took about 45 minutes but ranged from 20 to 60 minutes. Interviews were conducted until “information redundancy” was reached, after 18 interviews (Saunders et al., 2018). Ethical approval was provided by the NHS (REC reference 16/SC/0355). All participants signed a consent form specifically for the interview, which included explicit permission for the interview to be audio recorded and for quotations to be used anonymously in publications.

### Study Sample

All women were at least at 30 weeks’ gestation (early third trimester) at the time of the interview (*M* = 31.8 weeks, range 30.3–34.1). This timing was primarily selected so that women’s experiences across their pregnancies could be explored. One participant agreed to the interview but went into premature labor at 30 weeks before the interview took place; she still wished to take part, so she was interviewed on the postnatal ward 1 week after giving birth. The mean age of participants was 34 years (range 26–40) and mean BMI at 8 weeks’ gestation was 31 kg/m^2^ (range 22–44). All but one of the participants were born in the United Kingdom; one was born in Eastern Europe. One participant who was born in the United Kingdom identified as South Asian; the rest identified as White. All participants were employed at least part-time, although five participants had already begun maternity leave at the time of the interview. The majority had previously given birth (61%), and all participants were married or cohabiting. More than half (61%) of participants had university degrees or higher. Annual household income categories were reported as less than £20,000 (*n* = 1), £20,000-£40,000 (*n* = 8), £40,000-£60,000 (*n* = 6), and more than £60,000 (*n* = 3).

### Analysis

All interviews were audio-recorded, transcribed verbatim, and proofread for accuracy. Interview transcripts were thematically coded using NVivo, modeled after the guidance provided by King and Horrocks (2010). Given the semistructured nature of the interviews (and thus the discussion of a wide variety of topics during the interview that were not directly relevant to the questions at hand), sections within each transcript that were related to PA were given an initial deductive code, and inductive descriptive codes were assigned to each line within the initial code to capture its essence. Descriptive codes that shared common meanings were grouped together into interpretive codes and then into overarching themes. As we approached PA as a social practice, our coding scheme gave consideration to the ways in which PA was embedded within everyday lives and routines, as opposed to coding, for example, decontextualized “barriers” or “enablers” to PA. The analysis was an iterative process, involving discussion between the authors, and the original data were revisited at each stage to ensure the interpretive codes and themes were accurate representations. In our analysis, we paid close attention to themes emerging due to consistent experiences across participants, but also to variation of understandings and experiences, and we highlight such variation in our findings.

In the presentation of the results, the use of italics denotes direct quotations from participants. As there were no distinct thematic differences between those who were and were not diagnosed with GDM, we do not distinguish between these groups in our analysis and discussion.

## Results

### Leisure-Time PAs as a Way to Have “*Fun*” and “*Feel Better*”

Almost all women in this study described taking part in at least one form of leisure-time PA during their pregnancy. For many women, this included walking (for leisure) or going for long walks with their dog(s), while some took part in yoga, swimming, aqua natal classes, exercise classes (including barre, bootcamp, and group personal training sessions), strength/weight training, and/or running. These activities were described as things they “*enjoy*” and found to be “*fun*” or “*entertaining*.” In most cases, the leisure-time PAs in which participants took part during their pregnancies were activities they had enjoyed prior to pregnancy and continued for that reason:

*I love [lifting] weights. I got into it when I was getting married . . . weights was the best thing I ever found so to be able to still do that at 31 weeks pregnant . . . swimming I’ve always enjoyed . . . and then walking ‘cause . . . I just love picking up a map—I’m a geographer and [I love] picking up a map and going*, “*We’re gonna go here today and see what you find.*” *It’s doing exercise but it’s stuff I enjoy.*

Women identified a range of aspects of their selected activity/activities that they found especially pleasurable. For example, environmental aspects such as feeling the “*fresh air*” and “*see[ing] the birds*” enriched the experience of walking outside for leisure. Opportunities to socialize or do things “*together*” were also important components of some activities, such as going for walks with their partners, co-workers, or friends, going swimming with their mothers, or attending exercise classes with other pregnant or postnatal women.

Participants consistently emphasized the importance of leisure-time PA as a way to help them, as one participant put it, “*feel a million times better*” mentally and emotionally. “*To feel better*” was the predominant reason that women made intentional efforts to integrate PA into their pregnancies. They described the ways in which they used leisure-time PA to “*switch off*” and transform feeling “*hormonal*” into feeling “*better*,” “*relaxed*,” “*energized*,” and “*happier*”:

*[Physical activity is] good for the mum, I reckon—for yourself, just for your wellbeing and mentally as well. Like I find that if I feel a bit hormonal or I’m a bit moody or even tired, if I’ve gone and done a class or something I instantly feel much better afterwards. Like yeah, it might have been a tough class to get through but afterward actually I feel more energized.*

For some, “feeling better” related to the management of long-term mental health struggles. For example, one participant noted the importance of PA for managing her long-term anxiety, and another described walking as a way to combat feelings of loneliness and depression which had begun before her pregnancy: “*It made me feel better to kind of forget about those things and walk around and not be miserable and not think about all the bad things*.”

Several women who were the (working) mothers of young toddlers also identified their leisure-time PAs as their “*escape*,” allowing them to carve out time to do something for themselves away from their responsibilities as mothers and employees:

*When I do feel like a bit yucky like the yoga especially helps ‘cause there’s a lot of like relaxation in it and meditation and stuff, so if I feel terrible I go there because I don’t necessarily go for the postures . . . I just go there to just, you know, to relax or obviously—I do the postures but it’s my time out of like, just for me, so to speak.*

Participants also mentioned links between leisure-time PA and improved pregnancy outcomes, although these comments were rare. For example, several hoped that PA during pregnancy would result in a shorter labor or reduce the risks of complications, including GDM. There was little emphasis on being responsible for keeping active as a way of managing their weight or risk of GDM. In fact, women discussed their weight (many women had been identified as being at risk of GDM because of having a BMI ≥30) as being largely unmodifiable during pregnancy. The overall sense was that participants principally identified leisure-time PA as important for enjoyment and mental health:

*I think it’s a no-brainer that if you move more you’re less at risk [of things like gestational diabetes], but then as I said that’s not the only reason why I was moving about quite a lot. It’s because I do like moving about a lot and it’s because my mental health is better if I move a lot.*

### Negotiating PA as a Component of Everyday Practices and Roles

The ways in which participants’ everyday lives were structured varied day to day depending to a great extent on what was happening at work, with their partners, and with their children (for those who had them). For example, participants’ work schedules were not always identical from day to day, their partners sometimes worked out of town or did shift work, and their young children had bouts of illnesses (such as chicken pox or flu) that required around-the-clock care. These roles in their lives (employee, partner, and mother), and the constantly changing demands within them, strongly influenced how or whether leisure-time PA fitted into their lives on a given day, a given week, or in a given pregnancy. The interaction of these factors is apparent in two women’s descriptions of the logistical challenges that made leisure-time PA difficult to manage in this pregnancy:

*My partner works away Monday to Friday and he’s literally [only] home on the weekends. It’s just me and her [three-year-old daughter] during the week. I can only do so much . . . [In my first pregnancy] I used to work half past eleven until eight so quite long days, but I would like sort of go home and I could go for a walk, or go for a walk in the morning, and I used to do the antenatal classes as well. I can’t do that anymore ‘cause they don’t have childcare on a night so she hasn’t got anywhere to go, so it’s more restricted because I’m a parent.*

*I haven’t done any exercise classes or anything but that’s nothing to do with not wanting to do them. It’s just, with [partner] working shifts and having [3-year-old son], it’s really difficult to commit to anything*.

Some women described intentionally working PAs into their everyday routines, particularly if leisure-time PA was not feasible. For example, two women were offered parking spaces closer to the entrance of their work buildings because they were pregnant, but they purposefully did not utilize these and continued to park far away “*so I’ve got like at least a five minute walk to my desk on the morning*.” Others purposefully walked their dogs instead of delegating to a partner because “*I was conscious that I wasn’t doing other forms of exercise*.”

PAs were also a built-in requirement of their roles, particularly as employees and mothers. While most women did not have physically strenuous jobs, they noted that there were physical tasks of varying intensities associated with the kinds of work they were required to do. For example, one participant worked in a nursery which required “*a lot of standing, getting up and down with the children; they do like running round and games in the garden*”; another worked in retail which required being “*on me feet all the time, stocking shelves . . . general cleaning and stuff like that as well*.” Some noted that while they felt certain work-related physical tasks might not be appropriate to continue in later stages during pregnancy, it was often not practical to delegate the task to someone else:

*I’m still doing more lifting than I probably should but in a pub when it’s busy sometimes it’s impossible to wait for someone else to become free, like the glass trays have to be pulled out of the machine or put in and on a busy shift I just grit my teeth and do it.*

Looking after their own children was also physically demanding. Participants described the physical movement required to clean up after them, bathe them, carry them places, and noted that “*walking to and from his bedroom like five times with bucket loads of toys is exercise*.” Playing with their children was also an important component of regular life, which often involved PA outside:

*I go for walks at weekends, my son loves the outdoors so we’re always out and about . . . we’ve been to every forest in the north east and they all have like little toddler walks just a couple of miles or whatever so we tend to just do them and just out and about, really.*

*Taking him [son] outside and playing football on the field or even just like walking around a bit more on his scooter or his bike or whatever . . . just getting outside with him.*

Thus women identified their daily routines as revolving around their obligations within the home and as employees. These routines tended to limit their scope to be physically active, although some women negotiated time-spaces for daily activity, and others described being physically active within those roles.

### Negotiating Expectations That Pregnant Women Should “Slow Down”

Women consistently reported that throughout their pregnancies, those they encountered in their daily lives expected them to sit down and to slow down. Women described being encouraged to “*sit down*,” “*slow down*,” “*you just sit there*,” “*sit yourself down*,” “*get a cuppa and just sit down*,” “*have a seat*,” “*rest*,” “*take it easy*,” and “*put your feet up.*” Participants linked these comments to “*everyone*” or “*people*” as well as specific people in their everyday lives, including their partners/husbands, parents, and co-workers. For example, one participant described this exchange with her husband when discussing how she would spend a day off of work:

*I said*, “*Oh great, I can get this done and this done and this done . . .* ” *[Imitating her husband]* “*No, no, you can’t do that, you need to rest. No, no, you can’t do any of that.*” *I said to him*, “*I don’t have to sit all the time, I can do some things.*” “*No, no, no, no, no, have to rest, have to rest. Get the rest while you can.*”

Participants also reported being discouraged from PA. In the most extreme cases, several women described being barred from the gym because of their pregnant status and spoke of pregnant friends who stopped going to the gym because of “*the looks she was getting*.” However, disapproval of PA during pregnancy was not limited to exercise or high-intensity contexts; PA in everyday life, even of the lowest intensity, was also discouraged by those around them. For example, participants described being “*told off*” or “*getting wrong*” for tasks including standing, walking, vacuuming, scrubbing toilets, carrying shopping bags or laundry baskets, painting in the house, traveling (via train), bending down to reach low shelves, walking upstairs, moving or rearranging light furniture, visiting sites (by car) as part of their job, and picking up their toddlers:

*I get told an awful lot off older people for just you know standing or walking or picking [son] up, which I know like he’s heavier than what I’m allowed to pick up, but he’s my son and he needs picking up sometimes. What am I gonna do?*

While participants indicated that they interpreted others’ restrictions on their PA as “*caring*,” “*concern*,” and “*friendly advice*” intended to keep them from pushing themselves “*too much*,” they also found it “*annoying*,” particularly when it was not necessarily based on how they were feeling at the time. Participants specified that these comments started “*straight away*” once their pregnancy status was known (either by verbal disclosure or visibility of the bump) and that they were often incongruous with how capable women felt at a given time:

“*Oh don’t lift anything! Don’t move anything! Oh, you can’t do that!*” *. . . At work, people are like*, “*Oooh, don’t move that chair!*” “*Don’t climb the seven flights of stairs after the fire alarm’s gone off!*” *I’m like*, “*it’s just stairs.*”

Participants had several theories about where this expectation to sit down and slow down during pregnancy may have originated. Several participants used terms such as “*from the past*,” “*traditional*,” “*the dark ages*,” “*old wives’ tales*,” and “*Tudor times*” to suggest that the assumption that pregnant women should be doing nothing is outdated. Some speculated that their partners had interpreted their tiredness as evidence that they had done “*too much*,” and the encouragement to rest was therefore an attempt to prevent or mitigate the tiredness. Others suggested that the social perception of pregnancy as “*an illness*” was what prompted others to “*smother*” them and “*wrap [them] in cotton wool*”:

*It’s [pregnancy is] still treated a little bit like a special circumstance. A lot of people just don’t think it’s okay to work or do anything and it’s pretty much just laying on a couch, you know?*

However, participants generally strongly disagreed with the perception of pregnancy as a “*condition*” that automatically necessitated sitting and resting:

*This whole idea that pregnancy makes you weak—actually it’s one of the strongest things, you know, going through labor is one of the hardest things you’re ever gonna do. You need to be strong and fit to do that and you’re not gonna get that from putting your feet up and having a cup of tea and a slice of cake.*

Women negotiated this expectation to sit down and slow down in various ways throughout their pregnancies. In some instances, they rejected it (“*I just say ‘whatever’*”) and carried on doing what they were doing despite the expectations they encountered. In other cases, they complied with this expectation. This did not necessarily mean that they agreed with the suggestion to slow down and stop what they were doing, but, as one participant indicated, it was the path of least resistance:

*[When moving house] I was made to sit down and dictate where things went after a while.* “*You just sit there, you just—*” Interviewer: How did that make you feel? *By that point I think I’d been told off enough times that I just did what I was told ‘cause I was sick of it.*

In some cases, participants described a more strategic approach in which they carried on doing what they were told not to do out of the view of the person who told them to stop. For example, one participant described trying to get things done around the house without her husband (who had insisted she rest “*all the time*”) seeing what she was doing:

*Well [husband] was cutting the grass on Saturday and then I kept looking to see where he was and kept doing little things and then sneaking to do something and then coming back to the sofa [laughter].*

Similarly, two women shared their independent experiences of attending personal weight-training sessions that were held in (female) trainers’ homes (not the same trainer), specifically to avoid the social disapproval they had felt or experienced at the gym. While they described feeling freer to engage in the activities they felt were appropriate for their bodies without having to deal with disapproval in this environment, one commented that this phenomenon of doing activity during pregnancy in secret probably made it harder to normalize being active during pregnancy:

*There’s just not enough pregnant women doing it [physical activity], is there? And if they are doing it, they’re doing it at someone’s house where they’re a little bit hidden away.*

### Negotiating Physical Capability and PA

Changes in the physical experience of pregnancy were a central theme throughout women’s accounts. Most participants talked about feeling “*constant nausea*” and sometimes being sick, particularly in the first trimester. For most, this subsided during the second trimester before exhaustion, pain in the low back and/or the hips, breathlessness, and puffy feet caused discomfort in the third trimester:

*The first trimester was probably the hardest, just with the constant nausea and tired and lethargic, and I couldn’t really maintain what I would normally do . . . Second trimester then I started coming out of that, started feeling better, a bit more energy, felt like I could get back to myself, a little bit more which was great, I could pick up my exercise . . . The day my third trimester started I was like*, “*What is this new hell?*” *. . . I’ve got eight weeks left to go and I just, it’s just the slowing down, it I find quite a struggle for me . . . I’m a lot slower on my feet, and I can’t control it.*

The relationship between these physical experiences of pregnancy and PA was fluid and, at times, was bidirectional. For example, women talked about the ways in which their physical feelings interfered with their PA: the constantly impending sickness or extreme tiredness experienced by some in the first trimester made it difficult to even get out of bed or stray too far from home, breathlessness sometimes necessitated sitting at regular intervals, and pain in the hips impeded some activities. One participant described this in her third trimester:

*With this having pains in my hips now, I just can’t walk as far or like, when I’m cooking the tea, I can’t stand as long when I’m cooking it so I might have to have a quick sit-down while I’m waiting for water to boil.*

In these instances, the intensity of these feelings was enough to make PA of any kind unmanageable. At other times, however, when the physical feelings were less severe (although still present), PA provided some relief. For example, running helped alleviate nausea, exercise classes brought a “*boost*” of energy to combat extreme tiredness, and walking sometimes eased hip pain:

*[My hip pain] was worse on a morning when I got up because I had been lying all night and then once I like walked into work from the car park, the more movement I had the better . . . moving more has helped.*

Throughout this fluctuating relationship between PA and physical capability, participants consistently remarked that it was important to “*listen to the body*” to identify where the upper and lower limits for PA were. They emphasized that because the physical experience of pregnancy was so variable (from woman to woman and from day to day), it was up to each woman to identify what PA was suitable based on how it felt for them at a given moment in time: “*As long as you’re comfortable, do what you want, really. That’s what I would say. You know your own body. It’ll tell you when it’s had enough.*” This meant that if certain activities or intensities felt good to them at a given time, they could carry on despite what others’ expectations might be:

*I think everyone does seem to think I should be slowing down. But as I say, I know my body. If I can’t do it or if I’m too sore, then I won’t do it, and I’ll hold my hands up. But for now, I’ll just crack on*.

It also meant they needed to stay attuned to their body to know if they were doing “*too much*” and adjust the activity. In the case of leisure-time PA, this often meant reducing the intensity or switching the activity:

*When my body started getting sore after running . . . that’s the point where my body said, actually this is no longer comfortable anymore and I need to find something else to do. So I started strength training, which I really enjoyed.*

In the context of daily PA, such as work, this often resulted in delegating tasks or accepting help from others: “*[At work] I’m accepting peoples’ offers of help, whereas before I was like ‘No, no, I can do it’ . . . now I physically haven’t got the energy to do it.*”

## Discussion

This study has shown how pregnant women in this sample valued PAs, and the difficulties and complexities they faced in negotiating desired PA during pregnancy. For these women, PA was experienced as enjoyable and therapeutic, but we found that their opportunities to be active were constrained by their roles as workers and carers within the home, and by well-meaning exhortations from those around them to “slow down” or “sit down.” Nevertheless, women were often able to negotiate these constraints to integrate PAs within their lives, and felt able to attune their activity levels to the capabilities of their changing bodies.

The women in this sample articulated the ways in which their leisure-time PA practices brought them pleasure and enjoyment, helping them to “feel better.” Other studies have listed “feeling good” or “mental health” within the lists of “benefits,” “motivators,” or factors that “encouraged” PA during pregnancy in their samples (Cioffi et al., 2010; Denison et al., 2015; Leppanen et al., 2014; Tucker & Fouts, 2016; Weir et al., 2010). To our knowledge, only one other study has mentioned that “having fun” was a “reason” women exercised during their pregnancies (Bauer et al., 2018), but that study’s methodology (questionnaire) precluded further explorations of *how* or *why* certain activities were fun. In this sample, pleasurable elements of leisure-time PA included opportunities to be outdoors, to socialize, and (for mothers) to have time to themselves. Similarly, based on qualitative methods including in-depth semistructured interviews, E. V. Bennett et al. (2013) described the opportunity for social connections with other mothers-to-be as a critical component of participation in antenatal exercise classes and O’Brien et al. (2017) and Lloyd et al. (2016) described leisure-time PA among mothers of young children as a practice of “self-care” and a way to create “personal space” outside the obligations imposed by motherhood. Our findings also echo those of Fullagar (2008) in relation to a “playful care of the self” (p. 47) achieved by women recovering from depression through leisure practices, including a range of PAs that women experienced as invigorating, and of Morris et al. (2019) on the ways in which group walking was enjoyed by women. Thus, we identify an embodied and therapeutic sense of enjoyment as an important reason for PA to find a place in pregnant women’s lives.

However, PA was always negotiated within the circumstances and routines of women’s everyday lives, which constrained opportunities for leisure-time PA. Other literature on PA during pregnancy has consistently mentioned children, work, and family responsibilities as “barriers” to PA (Bauer et al., 2018; Cioffi et al., 2010; Connelly et al., 2015; Evenson et al., 2009; Flannery et al., 2018; Leppanen et al., 2014; Weir et al., 2010). Such findings are echoed by qualitative research showing that women, especially mothers of young children, feel that they are responsible for their family’s well-being, resulting in a lack of time, space, and energy to engage in PA for leisure (O’Brien et al., 2017). However, we also found that in some cases, PA was negotiated around everyday routines. Similarly, Guell et al. (2012) found that commuters’ decisions about their daily mode of travel to work were shaped by their everyday social worlds, for example, in relation to child care arrangements and day-to-day changes in work schedules. As they show, people negotiate the organization of daily routines, and may be able to tactically establish their own spaces for PA. It is also important to emphasize that women’s roles as mothers and employees often required them to move, thus building PA into their daily lives.

Encountering the exhortations of others to slow down and “sit down” was a striking theme in the data and women were obliged to negotiate such expectations throughout their pregnancies. “Lack of social support” has previously been identified as a “barrier” to PA during pregnancy (Denison et al., 2015; Evenson et al., 2009; Harrison et al., 2018), but the meaning of this has been unclear. Our findings suggest, in line with those of van Mulken et al. (2016), that this disapproval was repeatedly encountered and we show further that women responded tactically. While in some senses a “barrier” to PA, our data show that it is important to move beyond this simple construction; women often negotiated this surveillance to achieve the activities they wished to engage in, whether as part of everyday routines such as doing tasks around the house or undertaking more strenuous forms of exercise.

Participants’ accounts also highlighted the fluidity of the relationship between the physical experience of pregnancy and PA practices. At times, tiredness, back and hip pain, and nausea impeded PA; at other times (when these feelings were less severe), PA was a way “to feel better” and alleviate these discomforts. Others have shown that physical complaints of pregnancy can be a “barrier to” PA and are also alleviated by PA (a “benefit” or “enabler”) (Bauer et al., 2018; Denison et al., 2015; Weir et al., 2010). However, this “barrier/enabler” approach implies a static, oppositional relationship between PA and the physical experience of pregnancy. Our data suggest that this relationship is constantly in flux and is continuously negotiated. This is similar to the findings of D. L. Bennett (2017), whose qualitative data showed that women’s cycling practices during pregnancy were continuously negotiated in relation to physiological obstacles through “monitoring their comfort throughout and adapting, persevering, or when the pain was significant, pausing or abandoning the cycling altogether” (p. 442) while also noting that “cycling could offer forms of bodily relief” (p. 443). The relationship between bodily discomfort and PA was thus ambiguous for many women, and often renegotiated.

As their physical capabilities and experiences changed across pregnancy, participants emphasized the importance of “listening to the body” to identify what PAs and intensities were appropriate for them at a given time. In other studies, pregnant women have also referred to the value of “listening to the body” as a way to gauge the appropriateness of PA (Evenson et al., 2009; van Mulken et al., 2016). The meaning of “listening to the body” during pregnancy has been interpreted in multiple ways. It may represent a personalized alternative to scientific knowledge (Versteeg et al., 2018) and, in the case of pregnancy, a way to understand being pregnant as an embodied rather than a medical experience (Harper & Rail, 2012; van Mulken et al., 2016). However, “listening to the body” can be interpreted as a form of self-surveillance by which women are taking responsibility for monitoring their own pregnancies (Fredriksen et al., 2008), thus functioning as an extension of the medical gaze (Bessett, 2010). Our participants’ accounts were generally in line with the former suggestion, and they often used the term to indicate a degree of resistance to the expectations of others. Thus, the notion of “listening to the body” was a way of drawing on privileged (available to women but not to others) embodied experience that could be used by women to justify their participation in PA despite the concerns of those around them. Women could also have drawn on current biomedical advice that pregnant women should engage in PA and “listen to their bodies” and adapt activity accordingly (see Department of Health and Social Care, 2019), but, interestingly, neither advice from health professionals nor public health guidelines were referenced by women.

Our findings have several implications for understanding and promoting PA, particularly during pregnancy, in public health contexts. Importantly, in contrast to the emphasis that the biomedical literature places on PA in relation to concrete health outcomes (e.g., to reduce the risk of GDM), very few women talked about PA in those terms. Thus there was little evidence that women engaged in PA from a sense of responsibility (which is not to say that they did not adopt discourses of responsibility more generally). This suggests that framing PA within public health messages as a way to have “fun” and “feel better” might be more meaningful and thus more effective than emphasizing the importance of PA for longer term (physical) health outcomes, and also has the advantage of avoiding further responsibilizing pregnant women. We agree with Phoenix and Orr (2014) that strategies to promote or increase PA (during pregnancy) might “look beyond the usual suspects” of reduction of obesity and diabetes risk and “bring the notion of pleasure into the foreground” (p. 101), an approach also recommended by Fullagar (2003). This approach does not minimize the positive health effects that PA provides but allows other factors such as enjoyment to become a more central reason for being active (Tainio, 2019). Second, women should be supported to be active in ways that work for them during their pregnancies. This may include helping women identify activities they enjoy and things that make them feel good, as well as acknowledging (and accepting) that as the physical experience of pregnancy changes, different PAs may be more or less accessible at different times. Third, it is important for public health practitioners and midwives to be aware that pregnant women might encounter social disapproval of PA in their everyday lives, which may make it more difficult for them to respond to messages to “move more.” While broader societal changes are needed to shift maternal responsibility for the fetus and minimize responsibilizing discourses around “lifestyle” (Warin et al., 2012), it would be helpful for midwives and health professionals to emphasize the broad (i.e., not just health-related) value of PA during pregnancy to pregnant women themselves, and to their families.

The findings of this study should be interpreted in light of its limitations. First, this study’s sample was women who had been labeled as at risk of GDM during their pregnancy and participants were also predominantly White. Thus the findings may not necessarily be generalizable to pregnant women more broadly. Second, as the interviews were a component of a broader study about PA and GDM, participants may have placed particular (positive) emphasis on PA in their narratives. Finally, our reliance on interviews meant that we were only able to analyze participants’ accounts of their experiences of PA during pregnancy and other methods, such as participant observation, may have provided more insight into factors that could not be articulated by participants (Bernard, 2011).

## Conclusion

The women in our study, while acknowledging a link between PA and health, emphasized that activities such as walking and exercise classes were fun and helped them “feel better.” However, their gendered roles within the household and a public gaze that was concerned with the risks of PA for their fetus meant that women often had to negotiate opportunities for PA. Indeed, for at least some women, participation in PAs appeared to be a pleasurable, therapeutic, and transformative way to resist ideologies around pregnancy.

Our findings highlight the complexity of integrating and negotiating PA within everyday life during pregnancy. Most work that has aimed to understand PA during pregnancy has approached it in terms of “barriers,” “enablers,” and “attitudes,” which may have obscured a number of elements that underpin women’s PA practices. We suggest that women ought to be supported to be active in ways that they enjoy and that help them feel better during their pregnancies, with the acknowledgment that physical complaints and life circumstances might impede this at times, and that women may need to negotiate injunctions to “slow down.” These findings have implications for the design of any public health interventions aimed at increasing PA during pregnancy, particularly for women at high risk of GDM. Further research that uses more in-depth methods, such as ethnography, would be useful to deepen our understanding of women’s everyday experiences of PA during pregnancy.

## Supplemental Material

QHR_Supplemental_File – Supplemental material for “Sit Yourself Down”: Women’s Experiences of Negotiating Physical Activity During Pregnancy Click here for additional data file. Supplemental material, QHR_Supplemental_File for “Sit Yourself Down”: Women’s Experiences of Negotiating Physical Activity During Pregnancy by Janelle M. Wagnild and Tessa M. Pollard in Qualitative Health Research
