# Supplementary material for: “Sit Yourself Down”: Women’s Experiences of Negotiating Physical Activity During Pregnancy
Source: Qual Health Res. 2020 Mar 6;30(7):1072–82. doi: 10.1177/1049732320909103 (PMC7682526; doi:10.1177/1049732320909103)
Supplement: QHR_Supplemental_File – Supplemental material for “Sit Yourself Down”: Women’s Experiences of Negotiating Physical Activity During Pregnancy [file QHR_Supplemental_File.pdf]

Semi-structured interview guide

1. How would you describe what your pregnancy has been like so far?
2. In what ways have you found your lifestyle while pregnant has changed from your pre-pregnancy lifestyle?
3. Has there been anything about your experience during pregnancy that you didn't expect?
4. You were recruited to participate in this study because you had a risk factor for gestational diabetes. What does it mean to you to be 'at risk'? Were you diagnosed with gestational diabetes? Did knowing you were 'at risk' cause any changes in your lifestyle at all (regardless of diagnosis outcome)?
5. (For those diagnosed with gestational diabetes) Where did you get most of your information about what gestational diabetes is and how to manage it?
